# Trends and external causes of traumatic brain injury and spinal cord injury mortality in south China, 2014–2018: an ecological study

**DOI:** 10.1186/s12889-021-12225-2

**Published:** 2021-12-07

**Authors:** Xue-yan Zheng, Qian Yi, Xiao-jun Xu, Rui-lin Meng, Shu-li Ma, Si-li Tang, Hao-feng Xu, Ying-shan Xu, Yan-jun Xu, Yi Yang

**Affiliations:** 1 Guangdong Provincial Center for Disease Control and Prevention, 160 Qunxian Road, Panyu District, Guangzhou, Guangdong China; 2 Department of Epidemiology and Biostatistics, School of Public Health, Guangdong Pharmaceutical University, Guangzhou, Guangdong China; 3 School of Public Health, Southern Medical University, Guangzhou, Guangdong China

**Keywords:** Traumatic brain injury, Spinal cord injury, Mortality, External cause

## Abstract

**Background:**

The epidemiological evidence of traumatic brain injury (TBI) and spinal cord injury (SCI) mortality in mainland China is lacking. We aimed to assess the trends of TBI and SCI mortality, and their association with sex, age, location and external causes of injury in south China.

**Methods:**

Mortality data were derived from the Disease Surveillance Points (DSPs) system of Guangdong province between 2014 and 2018. We examined the trends in mortality with Cochran–Armitage trend test, and the association between the socio-demographic factors and the TBI and SCI mortality by using negative binomial models. Subgroup analysis was performed by stratifying the external causes of TBI and SCI.

**Results:**

The age-standardized TBI mortality remained relatively stable (from 11.6 to 15.4 per 100,000), while the SCI mortality increased by 148.3% from 2014 to 2018. Compared with females and urban residents, the adjusted mortality rate ratios of males and rural residents were 2.3 and 2.0 for TBI, and 2.2 and 4.6 for SCI, respectively. TBI and SCI mortality increased substantially with age. Motor vehicle crashes and falls were the leading causes of TBI mortality in residents aged under 75 years and over 75 years, respectively. Falls were the most important external cause for SCI death of all ages.

**Conclusions:**

Being male, rural and elderly residents are at higher risk of dying from TBI and SCI. The substantial burden of TBI and SCI caused by road traffic crashes and falls has called for the urgent need to improve injury prevention, pre-hospital aid, hospital treatment and recovery.

**Supplementary Information:**

The online version contains supplementary material available at 10.1186/s12889-021-12225-2.

## Introduction

Traumatic brain injury (TBI) and spinal cord injury (SCI) are injuries of the brain and spinal cord, leading to the functional alteration or pathological changes [1, 2]. Both the TBI and SCI are significant global public health issues which contribute to the excessive health-care and economic burden because of the loss of productivity and increased health-care costs [3]. In 2016, there were 27.1 million incident cases of TBI and 0.9 million incident cases of SCI globally, with the age-standardized incidence rates of 369 per 100,000 population for TBI and 13 per 100,000 for SCI [4]. In the developed countries such as the United States, TBI was associated with approximately 2.5 million emergency department visits, 282,000 hospitalizations, and 56,000 deaths in 2013 [5]. In China, the TBI-related mortality remained relatively high between 2006 and 2013, ranging from 13.0 to 17.1 per 100,000 population [6].

Understanding the patterns, causes, and trends of TBI and SCI is critical for identifying the risk factors, developing prevention measures, and evaluating interventions and policies at local, national, and global levels. Unfortunately, few studies have addressed the epidemiological characteristics of TBI and SCI morbidity and mortality aside from the data derived from developed countries [7–9]. Well-conducted epidemiological studies documenting the TBI and SCI are scarce in low- and middle-income countries, including China. Only three epidemiological studies on TBI and SCI have been conducted among outpatient clinics and/or inpatient departments at the local hospitals, which cannot be extrapolated to the provincial population [10–12]. These studies mainly reported the burden of injury when stratified by the causes of injury (i.e., self-harm, road injuries, and falls); however, the longevity of effects of TBI and SCI has not been well clarified.

Notwithstanding the huge burden, neither TBI nor SCI has been sufficiently addressed by medical researchers and policy-makers in developing countries. To address the knowledge gap, we have conducted an ecological study to determine the trends in TBI and SCI mortality, and the differences stratified by the geographic location (urban vs. rural), sex, age, and the external cause of TBI/SCI in Guangdong province between 2014 and 2018. Our findings might provide the rationale for implementing measures or prevention interventions for reducing deaths associated with TBI and SCI.

## Methods

### Data sources

The main data set of TBI and SCI in Guangdong province was derived from Disease Surveillance Points (DSPs), the Chinese Center for Disease Control and Prevention (CDC) Cause of Death Reporting System between 2014 and 2018.

Since 2006, Guangdong province initiated the death surveillance under the direction of Chinese CDC, consisting of eight DSPs. Each surveillance point represented a single district (urban area) or county (rural area), covering all residents of these geographic areas [13]. Since 2013, the Chinese government has introduced a web-based approach to report death cases using the DSPs system [14, 15], which remarkably improved the timeliness of data reporting. Meanwhile, the number of surveillance points increased to 28, rendering the data sets provincially representative. In 2015, the number of surveillance points further increased to 129, covering all counties and districts across Guangdong province [16].

We have adopted the data from 28 national DSPs between 2014 and 2018 for analysis in light of the improved data quality and the major updates of DSPs system since 2013. Duplicate records of death were removed. An internal data verification system evaluated the timeliness, completeness, and accuracy of data reporting, and the statistical measures (e.g., the standard United Nations Age–Sex Accuracy Index) were employed to monitor the data quality [17]. The completeness of death surveillance was estimated empirically as reported by Adair and Lopez [18]. Details of the internal data verification and the completeness of death surveillance are shown in E-Table 1.

### Definition of traumatic brain injury and spinal cord injury

Deaths related to injury were reported by experienced clinicians from the local hospitals, or the forensic physicians from the police departments. Public health physicians were trained for coding the cause of death by using the International Classifiers of Diseases-10 (ICD-10) codes. TBI was defined according to the ICD-10 codes, as recommended by the US CDC [19, 20]—namely, S01.0–S01.9, S02.0, S02.1, S02.3, S02.7–S02.9, S04.0, S06.0–S06.9, S07.0, S07.1, S07.8, S07.9, S09.7 − S09.9, T01.0, T02.0, T04.0, T06.0, T90.1, T90.2, T90.4, T90.5, T90.8, and T90.9. The SCI was identified based on the ICD-10 codes which consisted of S12.0-S12.2, S12.7, S12.9, S13.0, S13.1, S13.4, S14.0, S14.1, S22.0, S22.1, S23.0, S23.1, S24.0, S32.0-S32.2, S32.7, S33.0, S33.1, S33.5, S33.7, S34.0, S34.1, T08.0, T09.3, T91.1 and T91.3 [21]. Based on the published studies and ICD-10 codes [22], we have classified the underlying external causes of TBI and SCI deaths into four main categories: 1) motor vehicle crashes, 2) falls, 3) struck by and against, and 4) all other minor categories combined. To detail the subgroups of TBI and SCI deaths from motor vehicle crashes, we reported the results by the road user category. Road users were classified into five categories according to the ICD-10 injury matrix as recommended by the US CDC [23]: vehicle occupant, motorcyclist, pedal cyclist, pedestrian, and all other minor categories combined (Table 1).

**Table 1:** ICD codes for TBI, SCI, external causes and road user categories

	ICD codes
**Injury**	
TBI (Traumatic brain injury)	S01.0–S01.9, S02.0, S02.1, S02.3, S02.7–S02.9, S04.0, S06.0–S06.9, S07.0, S07.1, S07.8, S07.9, S09.7 − S09.9, T01.0, T02.0, T04.0, T06.0, T90.1, T90.2, T90.4, T90.5, T90.8, and T90.9
SCI (Spinal cord injury)	S12.0-S12.2, S12.7, S12.9, S13.0, S13.1, S13.4, S14.0, S14.1, S22.0, S22.1, S23.0, S23.1, S24.0, S32.0-S32.2, S32.7, S33.0, S33.1, S33.5, S33.7, S34.0, S34.1, T08.0, T09.3, T91.1 and T91.3.
**External causes for TBI and SCI**	
Motor vehicle crashes	V30–V79 [.4–.9], V81.1, V82.1, V83–V86 [.0–.3], V20–V28 [.3–.9], V29 [.4–.9], V12–V14 [.3–.9], V19 [.4–.6], V02–V04 [.1, .9], V09.2, V80 [.3–.5], V87 [.0–.8], V89.2, X82, Y03, Y32
Struck by and against	W20–W22, W50–W52, X79, Y00, Y04, Y29, Y35.3
Falls	W00–W19, X80, Y01, Y30
All others	the remaining ICD-10 codes for all other causes
**Road users categories**	
Vehicle occupant	V30 − V79 [.4 − .9], V81.1, V82.1, V83 − V86 [.0 − .3]
Motorcyclist	V20 − V28 [.3 − .9], V29 [.4 − .9]
Pedal cyclist	V12 − V14 [.3 − .9], V19 [.4 − .6]
Pedestrian	V02 − V04 [.1, .9], V09.2
All others	V80 [.3 − .5], V87 [.0 − .8], V89.2

### Statistical analyses

There have been major changes in the sampling methods in the DSPs database since 2013. The six DSPs have been expanded to 28 surveillance points after 2013, rending the database representative of Guangdong province. To minimize the bias associated with these changes, we analyzed the mortality data from the same 28 surveillance points between 2014 and 2018. We calculated the overall and cause-specific age-standardized mortality. The direct standardization method was adopted to adjust for the population structure. The census population in 2010 was used as the reference population since it has been the latest national census including the population number and proportion (with the results being previously published by National Bureau of Statistics) [24]. The 95% confidence intervals (95%CIs) of mortality rates were calculated. Because of the substantial age variations in TBI and SCI morbidity and mortality in previous literature reports [7], we stratified the age based on the following schemes: 0–4 years, 5–14 years, 15–24 years, 25–44 years, 45–64 years, 65–74 years, and 75 years or greater. We calculated the sex-, age-, location-, and cause-specific death rates to inform policy-making priorities. The Cochran–Armitage trend test was used to examine the significance of the trends in mortality [6]. Subgroup analysis was conducted by comparing the strata of location (urban/rural), sex, age, and external causes of TBI. Because of the over-dispersion of TBI/SCI-related deaths, we used multivariate negative binomial models to explore the associations between deaths and socio-demographic factors (location, sex, age, year) [25]. Mortality rate ratios (MRRs) and the corresponding 95% CIs were adopted to quantify the strength of associations.

All statistical analyses were performed with SAS software 9.4 (SAS Institute, Inc. Cary, NC USA). The threshold for statistical significance was set at 0.05 for the *P* value.

## Results

### Surveillance sample characteristics

Between 2014 and 2018, the population from the 28 DSPs ranged from 22.4 million to 24.6 million, covering one-fourth of the total population in Guangdong province. Males accounted for 51.0 to 51.5% of the surveillance sample, respectively. The proportion of urban residents increased from 43.7 to 44.7%. There was a trend towards a progressive increase in the proportion of the elderly (9.7% ~ 11.6%). Further details are demonstrated in E-Table 2.

### Overall trends of TBI and SCI mortality

During the study period, 16,169 TBI- and 749 SCI-related deaths were captured by the DSPs. The total age-standardized TBI mortality was 12.9 per 100,000 population (SE = 0.1), ranging from 11.6 per 100,000 population in 2014 to 11.9 per 100,000 population in 2018. The total age-standardized SCI mortality was 0.5 per 100,000 population (SE = 0.02), with an increase from 0.3 per 100,000 population in 2014 to 0.7 per 100,000 population in 2018 (Table 2). After adjusting for the socio-demographic factors, TBI mortality remained mostly unchanged (Table 3), while SCI mortality increased by 258% in 2015 (Table 4).

**Table 2:** Mortality rates of TBI and SCI per 100,000 population when stratified by the geographic location, sex, age group, and external cause of injury in Guangdong, 2014–2018

Characteristics	2014		2015		2016		2017		2018		Total		***Z***^**b**^	***P***
	Rate^**a**^	SE	Rate^**a**^	SE	Rate^**a**^	SE	Rate^**a**^	SE	Rate^**a**^	SE	Rate^**a**^	SE		
**TBI**	11.6	0.2	15.4	0.3	12.3	0.2	13.2	0.2	11.9	0.2	12.9	0.1	14.2	< 0.001
**Sex**														
Male	16.4	0.4	22.7	0.4	17.2	0.4	17.8	0.4	16.3	0.4	18.1	0.2	31.4	< 0.001
Female	6.5	0.2	7.9	0.3	7.2	0.3	8.5	0.3	7.4	0.3	7.5	0.1	−23.3	< 0.001
**Age group (year)**														
0 ~ 4	5.0	0.6	7.5	0.8	3.8	0.5	4.5	0.6	4.2	0.5	5.0	0.3	18.2	< 0.001
5 ~ 14	2.8	0.3	2.5	0.3	2.6	0.3	2.3	0.3	2.1	0.3	2.5	0.1	12.1	< 0.001
15 ~ 24	9.1	0.5	11.2	0.5	8.6	0.5	7.9	0.5	5.8	0.4	8.5	0.2	51.6	< 0.001
25 ~ 44	8.9	0.4	12.2	0.4	8.0	0.3	7.8	0.3	7.2	0.3	8.8	0.2	53.9	< 0.001
45 ~ 64	14.5	0.5	21.2	0.6	15.5	0.5	16.9	0.5	15.5	0.5	16.7	0.2	9.7	< 0.001
65 ~ 74	23.6	1.3	33.3	1.6	30.5	1.5	31.6	1.5	29.1	1.4	29.6	0.7	−15.0	< 0.001
≥ 75	48.7	2.2	52.4	1.9	66.9	2.6	87.4	3.0	80.0	2.9	67.1	1.1	−79.7	< 0.001
**Location**														
Urban	8.0	0.3	8.0	0.3	8.1	0.3	9.0	0.3	8.2	0.3	8.2	0.1	−11.7	< 0.001
Rural	15.1	0.4	23.0	0.4	16.6	0.4	17.6	0.4	15.8	0.3	17.6	0.2	25.3	< 0.001
**External causes**														
MVC	6.9	0.2	9.6	0.2	7.4	0.2	7.4	0.2	6.5	0.2	7.6	0.1	36.7	< 0.001
Falls	3.4	0.1	4.6	0.1	4.1	0.1	5.0	0.1	4.7	0.1	4.4	0.1	−49.6	< 0.001
Struck by/against	0.7	0.1	0.4	0.0	0.3	0.0	0.4	0.0	0.2	0.0	0.4	0.0	59.5	< 0.001
Others	0.5	0.1	0.8	0.1	0.5	0.1	0.5	0.0	0.5	0.1	0.6	0.0	20.5	< 0.001
**SCI**	0.3	0.0	0.5	0.0	0.5	0.1	0.7	0.1	0.7	0.1	0.5	0.0	−52.5	< 0.001
**Sex**														
Male	0.3	0.1	0.6	0.1	0.5	0.1	0.7	0.1	0.7	0.1	0.5	0.0	−38.9	< 0.001
Female	0.3	0.1	0.4	0.1	0.5	0.1	0.6	0.1	0.7	0.1	0.5	0.0	−35.3	< 0.001
**Age group (year)**														
0 ~ 4	0.0	0.0	0.0	0.0	0.0	0.0	0.1	0.1	0.0	0.0	0.0	0.0	−5.3	< 0.001
5 ~ 14	0.0	0.0	0.0	0.0	0.0	0.0	0.0	0.0	0.0	0.0	0.0	0.0	11.6	< 0.001
15 ~ 24	0.0	0.0	0.0	0.0	0.0	0.0	0.0	0.0	0.1	0.1	0.0	0.0	−3.5	< 0.001
25 ~ 44	0.1	0.0	0.1	0.0	0.1	0.0	0.2	0.1	0.2	0.1	0.1	0.0	−10.8	< 0.001
45 ~ 64	0.2	0.1	0.2	0.1	0.5	0.1	0.7	0.1	0.4	0.1	0.5	0.0	−14.3	< 0.001
65 ~ 74	0.7	0.2	1.2	0.3	0.6	0.2	1.0	0.3	2.0	0.4	1.1	0.1	−19.1	< 0.001
≥ 75	5.4	0.7	5.2	0.6	9.1	0.9	11.9	1.1	12.8	1.1	8.9	0.4	−48.4	< 0.001
**Location**														
Urban	0.3	0.1	0.3	0.1	0.5	0.1	0.7	0.1	0.7	0.1	0.5	0.0	−40.0	< 0.001
Rural	0.3	0.1	0.7	0.1	0.5	0.1	0.7	0.1	0.8	0.1	0.6	0.0	−34.6	< 0.001
**External causes**														
MVC	0.1	0.0	0.1	0.0	0.1	0.0	0.1	0.0	0.1	0.0	0.1	0.0	−8.8	< 0.001
Falls	0.2	0.0	0.2	0.0	0.3	0.0	0.4	0.0	0.4	0.0	0.3	0.0	−39.0	< 0.001
Others	0.1	0.0	0.1	0.0	0.2	0.0	0.2	0.0	0.3	0.0	0.2	0.0	−36.4	< 0.001

*SE* Standard error, *TBI* Traumatic brain injury, *SCI* Spinal cord injury, *MVC* Motor vehicle crash

^a^Mortality rates for total and subgroup traumatic brain injury (except for the 7 age groups) were age-standardized based on the population of China in 2010

^b^Mortality trends from 2006 to 2013 were examined using the Cochran–Armitage trend test

**Table 3:** Associations between traumatic brain injury mortality and the socio-demographic variables, estimated with the multivariate negative binomial regression **model** (Guangdong, 2014–2018)

Socio-demographic variable	TBI mortality by external cause of injury												Total TBI mortality		
	Motor vehicle crashes			Falls			Struck by/against			All others					
	***MRR***	95%***CI***		***MRR***	95%***CI***		***MRR***	95%***CI***		***MRR***	95%***CI***		***MRR***	95%***CI***	
		Lower	Upper		Lower	Upper		Lower	Upper		Lower	Upper		Lower	Upper
**Sex (reference = female)**															
Male	2.5*	2.1	3.0	2.1*	1.8	2.5	2.2*	1.2	3.9	2.1*	1.3	3.3	2.3*	2.0	2.6
**Age (reference = 25 ~ 44 years)**															
0 ~ 4	0.5*	0.4	0.7	1.0*	0.8	1.4	0.6*	0.2	1.6	0.4*	0.2	1.1	0.6*	0.5	0.8
5 ~ 14	0.3*	0.2	0.3	0.3*	0.2	0.4	0.3*	0.1	1.0	0.2*	0.1	0.5	0.3*	0.2	0.3
15 ~ 24	0.9*	0.7	1.3	0.8*	0.6	1.1	0.7*	0.2	1.9	0.7*	0.1	1.5	0.9*	0.7	1.1
45 ~ 64	1.8*	1.3	2.4	2.8*	2.1	3.7	1.6*	0.6	4.5	1.7*	0.7	3.9	1.9*	1.6	2.4
65 ~ 74	3.0*	2.2	4.0	6.9*	5.2	9.1	1.6*	0.6	4.5	3.7*	1.6	8.6	3.8*	3.0	4.8
≥ 75	3.6*	2.7	4.9	31.0*	23.3	41.2	3.7*	1.3	10.5	8.8*	3.8	20.8	10.4*	8.3	13.0
**Location (reference = urban)**															
Rural	2.5*	2.1	2.9	1.3*	1.1	1.5	2.9*	1.6	5.0	2.6*	1.6	4.3	2.0*	1.8	2.3
**Year (reference = 2014)**															
2015	1.3*	1.0	1.6	1.2*	1.0	1.6	0.6*	0.3	1.5	0.8*	0.4	1.6	1.2*	1.0	1.4
2016	1.1*	0.8	1.4	1.0*	0.8	1.2	0.5*	0.2	1.1	0.6*	0.3	1.3	1.0*	0.8	1.2
2017	1.1*	0.9	1.5	1.2*	1.0	1.6	0.6*	0.2	1.4	0.6*	0.3	1.3	1.1*	0.9	1.3
2018	0.9*	0.7	1.2	1.1*	0.9	1.5	0.4*	0.1	0.9	0.6*	0.3	1.3	1.0*	0.8	1.2

*MRR* Mortality rate ratio, adjusted for location, sex, age group and year, *CI* Confidence interval, *MRR* Mortality rate ratio, *TBI* Traumatic brain injury

*P < 0.05

**Table 4:** Associations between spinal cord injury mortality and the socio-demographic variables, estimated with the multivariate negative binomial regression model (Guangdong, 2014-2018)

Socio-demographic variable	SCI mortality by external cause of injury									Total SCI mortality		
	Motor vehicle crashes			Falls			Others					
	***MRR***	95%***CI***		***MRR***	95%***CI***		***MRR***	95%***CI***		***MRR***	95%***CI***	
		Lower	Upper		Lower	Upper		Lower	Upper		Lower	Upper
**Sex (reference = female)**												
Male	5.4*	1.4	23.0	2.5*	1.2	5.1	1.3*	0.4	4.7	2.2*	1.1	4.7
**Age (reference = 25 ~ 44 years)**												
0 ~ 4	0.0*	0.0	0.4	0.0*	0.0	0.0	0.0*	0.0	0.0	0.0*	0.0	0.1
5 ~ 14	0.1*	0.0	1.1	0.0*	0.0	0.0	0.0*	0.0	0.0	0.1*	0.0	0.3
15 ~ 24	0.0*	0.0	0.2	0.0*	0.0	0.0	0.7*	0.1	6.6	0.1*	0.0	0.4
45 ~ 64	1.5*	0.2	13.2	4.7*	1.9	11.9	4.6*	0.8	27.3	3.2*	1.0	10.1
65 ~ 74	3.8*	0.4	38.1	17.3*	6.8	44.8	23.9*	4.0	146.3	13.9*	4.2	46.0
≥ 75	3.1*	0.3	29.9	238.0*	86.8	665.6	188.5*	33.7	1064.5	157.5*	47.8	523.6
**Location (reference = Urban)**												
Rural	6.8*	1.8	31.4	1.2*	0.6	2.2	2.4*	0.6	10.0	4.6*	2.0	11.4
**Year (reference = 2014)**												
2015	2.6*	0.3	20.5	1.9*	0.7	5.3	2.9*	0.4	28.1	3.6*	1.2	11.4
2016	1.2*	0.2	9.3	1.8*	0.7	5.0	1.5*	0.3	8.6	1.0*	0.3	2.9
2017	2.0*	0.2	19.5	2.7*	1.0	7.2	2.5*	0.4	14.5	2.8*	0.9	9.4
2018	4.8*	0.6	44.7	4.1*	1.5	11.2	2.3*	0.4	13.9	2.0*	0.7	5.6

*MRR* Mortality rate ratio, adjusted for location, sex, age group and year, *CI* Confidence interval, *MRR* Mortality rate ratio, *SCI* Spinal cord injury

*P < 0.05

### Sex difference

Compared with females, males had a 1.4-times greater risk of dying from TBI (Table 2; E-Fig. 1A). TBI mortality for both males and females showed a similar pattern of change over time. After adjusting for the socio-demographic factors and year, the risk was 2.3 times higher in males dying from TBI than in females. The adjusted male–female MRR was 2.5 for TBI from motor vehicle crashes, 2.1 from falls, 2.2 from struck by or against and 2.1 from other causes, respectively (Table 3). Males appeared to have similar risks of dying from SCI compared with females (0.5 per 100,000 population in males versus 0.5 per 100,000 population in females) (Table 2; E-Fig. 2A); however, there was a 1.2-fold higher risk of dying from SCI in males than in females, after adjusting for the socio-demographic factors and year. The adjusted MRR for males was 2.5 for SCI from falls, but reached 5.4 for SCI from motor vehicle crashes (Table 4).

### Variations across age groups

The TBI and SCI mortality varied substantially among different age groups (E-Figs. 1B and 2B). Children aged 5–14 years had the lowest TBI mortality (2.5 per 100,000 population), while adults aged 75 years or greater had the highest mortality (67.1 per 100,000 population). Similar trends also applied for SCI (Table 2). For people aged under 75 years, TBI and SCI mortality did not differ considerably within each of the age strata. However, the population aged 75 years or greater yielded markedly higher TBI and SCI deaths (48.7 and 5.4 per 100,000 people) in 2014 and (80.0 and 12.8 per 100,000) in 2018 (Table 2).

Even after adjusting for the socio-demographic factors and year, there remained notable variations in TBI and SCI mortality across different age groups. Compared with the population aged 25-44 years, TBI mortality from motor vehicle crashes, striking, and other causes increased by 261, 267 and 783% in the population aged 75 years or greater, respectively. The adjusted MRR reached 31.0 and 238.0 for TBI and SCI death associated with falls in population aged 75 years or greater as compared with the population aged 25-44 years (Table 3, Table 4).

### Urban–rural difference

Rural residents had higher TBI mortality rates than urban residents. The age-standardized rural-urban mortality ratio for TBI and SCI was 2.1 and 1.2, respectively (Table 2). After adjusting for sex, age and year, rural residents remained at a greater risk of dying from TBI (MRR: 2.0, 95% CI: 1.8–2.3) and the TBI due to external causes of motor vehicle crashes (MRR: 2.5, 95% CI: 2.1–2.9), falls (MRR: 1.3, 95% CI: 1.1–1.5), and striking (MRR: 2.9, 95% CI: 1.6–5.0), when compared with the urban residents (Table 3). Similarly, an increased risk was found in rural residents for SCI mortality (MRR: 4.6, 95% CI: 2.0–11.4), which mainly resulted from motor vehicle crashes (MRR: 6.8, 95% CI: 1.4–31.4) (Table 4). TBI age-standardized mortality rates followed similar patterns for both urban and rural residents (E-Fig. 1C), while SCI mortality varied considerably in urban and rural residents (E-Fig. 2C).

### External causes of TBI-related deaths

**Fig. 1:**
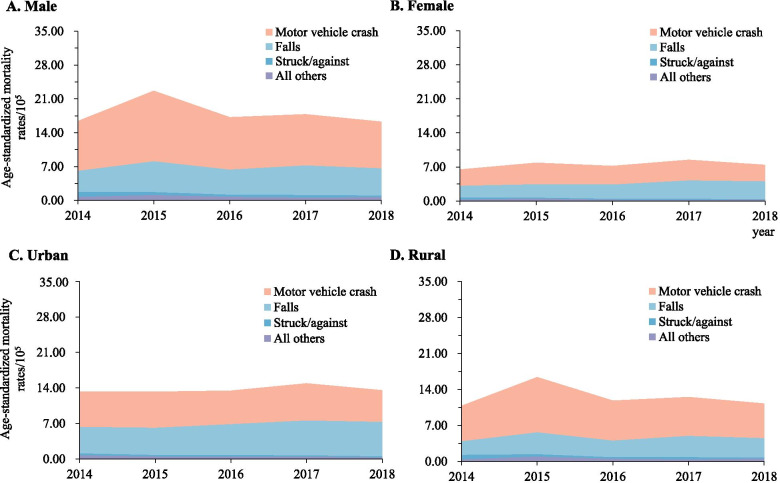
Mortality rates of traumatic brain injury when stratified by urban/rural location, sex, and external cause of injury (Guangdong, China, 2014–2018). **A** Male; **B** Female; **C** Urban area; **D** Rural areas. Mortality rates were age-standardized based on the population of China in 2010

**Fig. 2:**
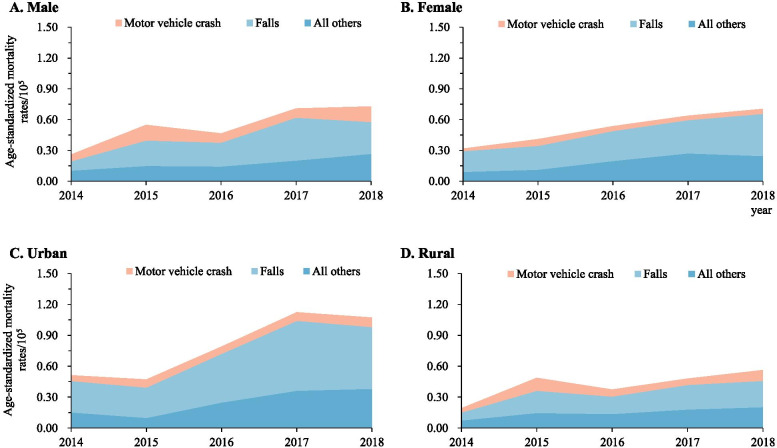
Mortality rates of spinal cord injury when stratified by urban/rural location, sex, and external cause of injury (Guangdong, 2014-2018). **A** Male; **B** Female; **C** Urban areas; **D** Rural areas;. Mortality rates were age-standardized based on the population of China in 2010

Between 2014 and 2018, motor vehicle crashes and falls were consistently the leading causes of TBI- and SCI-related mortality in both urban and rural residents, and in both males and females (Figs. 1 and 2). The difference in cause-specific TBI and SCI mortality was substantial when comparing urban with rural areas, and when comparing males with females.

**Fig. 3:**
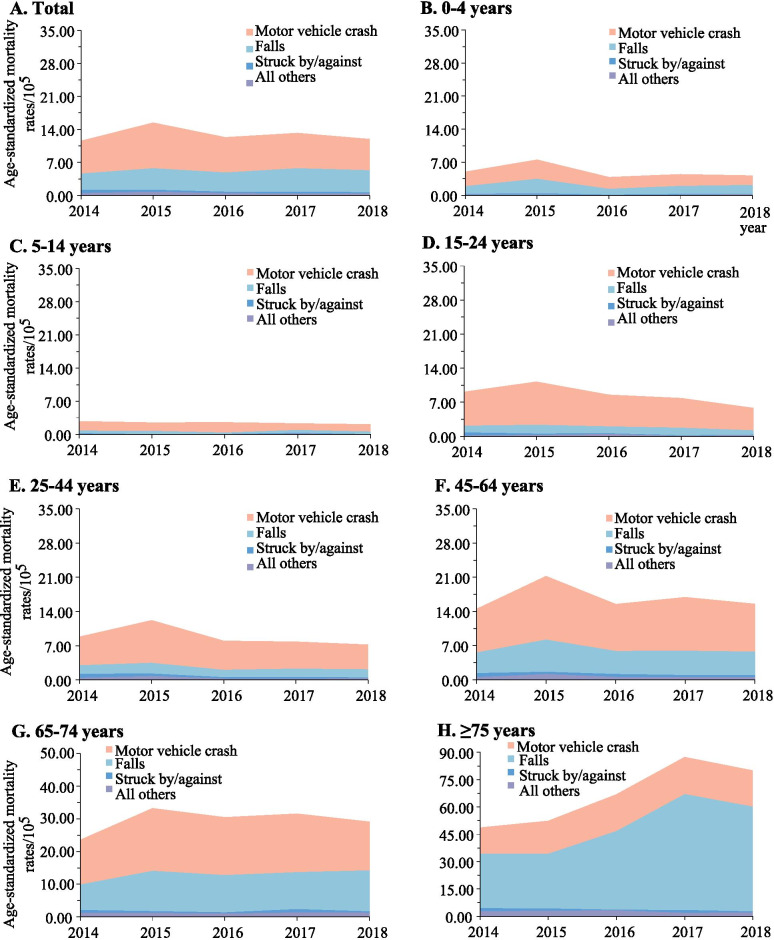
Mortality rates of traumatic brain injury when stratified by age group and external cause of injury (Guangdong, 2014–2018)

**Fig. 4:**
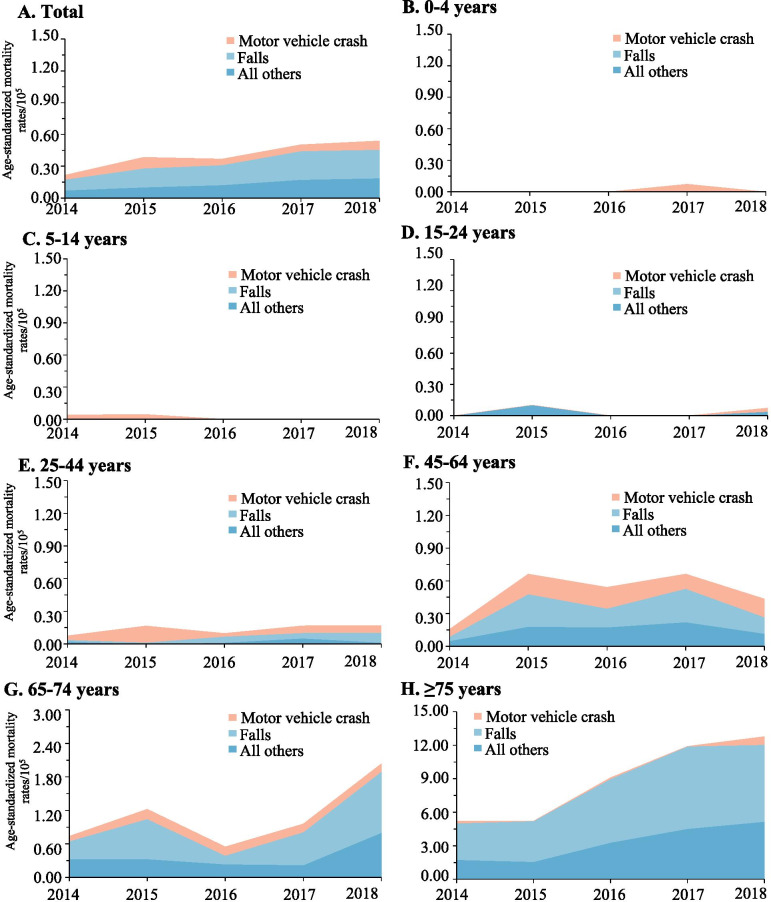
Mortality rates of spinal cord injury when stratified by age group and external cause of injury (Guangdong, 2014–2018)

The spectrum of the causes of TBI mortality varied across different age groups. Motor vehicle crashes were the leading cause in the population aged 75 years or lower, while falls dominated the cause in adults aged 75 years or greater (Fig. 3). Among the population aged 15 years or greater, the contribution of falls to the total TBI mortality increased progressively as the age increased. By contrast, falls dominated the cause of SCI in all age groups (Fig. 4).

**Fig. 5:**
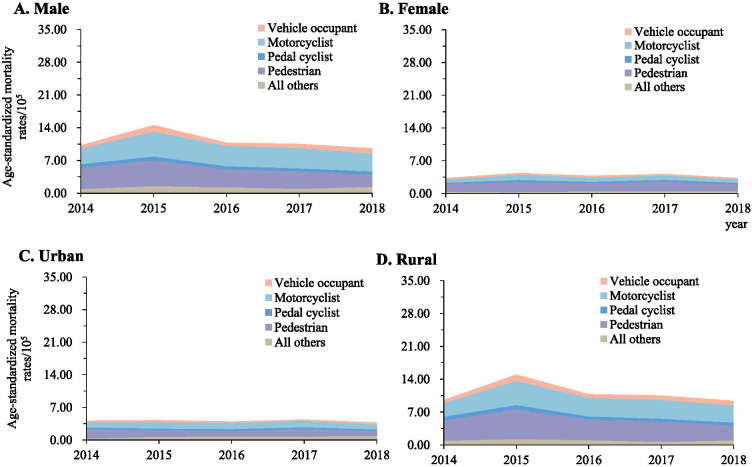
Mortality rates from traumatic brain injury due to motor vehicle crashes by urban/rural location, sex, and road user category (Guangdong, 2014–2018). **A** Male; **B** female; **C** Urban areas; **D** Rural areas. Mortality rates were age-standardized based on the population of China in 2010

Pedestrians were the most vulnerable road users for TBI deaths attributed to motor vehicle crashes in both urban and rural areas, and in males and females (Fig. 5). For SCI mortality, motorcyclist and pedestrians dominated the causes of motor vehicle crashes (E-Fig. 3). Compared with females and urban residents, higher risks of TBI mortality among vehicle occupant, motorcyclists, pedal cyclist and pedestrian were found in males and rural residents (Table 3).

**Fig. 6:**
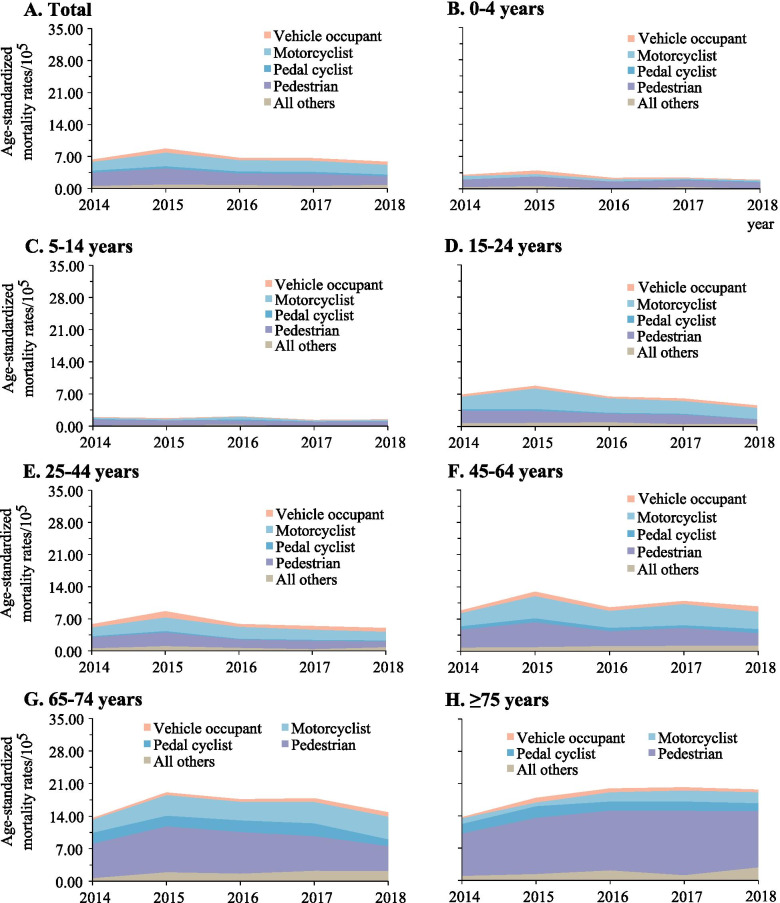
Mortality rates from traumatic brain injury due to motor vehicle crashes when stratified by the age group and road user category (Guangdong, 2014–2018)

TBI mortality varied substantially among different age groups. Pedestrians aged 14 years or lower and those aged 65 years or greater dominated the TBI mortality owning to motor vehicle crashes. For the 15–24, 25–44, and 45–64 years age groups, motorcyclist deaths were the most vulnerable populations of TBI mortality due to motor vehicle crashes, accounting for 47.4, 37.8 and 38.0% of the total TBI mortality, respectively (Fig. 6). Pedestrians accounted for more than 30.0% of the total SCI mortality from motor vehicle crashes in adults aged under 65 years and those aged 75 years or greater. While motorcyclists for SCI mortality caused by motor vehicle crashes accounted for 34.3% of the total death between 2014 and 2018, with a greater contribution among adults aged 65 and 74 years.

## Discussion

### Principal findings

Characterizing the causes of TBI or SCI mortality may help understand the effect of injury prevention programs. We demonstrated the socio-demographic patterns, trends, and external causes of TBI and SCI mortality in south China. We have revealed the contrasting trends of changes in the overall age-standardized mortality of TBI and SCI, and the variation in TBI and SCI age-standardized mortality stratified by locations, sex, and age. We showed that motor vehicle crashes dominated the TBI mortality among residents aged under 75 years, that pedestrians were the vulnerable population of TBI deaths from motor vehicle crashes in children and the elderly, and that TBI mortality increased in adults aged 45–74 years old.

### TBI and SCI mortality

The overall TBI mortality in Guangdong, ranging between 11.6 and 15.4 per 100,000 population between 2014 and 2018, was comparable to that reported in a number of countries, including mainland China (13.0-17.1 per 100,000 population) [6], the US (17.0 per 100,000 population) [5], Europe (11.7 per 100,000 population) [26], and Germany (11.5 per 100,000 population) [27]. In contrast to the decreasing trend in the US [28], Austria [29] and Canada [30], the TBI mortality in Guangdong province increased slightly in 2015, followed by a decrease between 2016 and 2018. The overall SCI mortality in Guangdong (0.5 per 100,000 population) was higher than in Austria between 2002 and 2012 (2.7 per 100,000 population) [31]. Given the potential differences in the population structure, culture, study duration, data collection methods and study definition [32, 33], the TBI and SCI mortality across countries and regions should be interpreted with caution. However, the data indicated that both TBI and SCI have become a significant public health issue in Guangdong province.

In line with previous studies [6, 7, 34], we have identified high TBI and SCI mortality in males, rural residents, and older adults. Notably, there was an increased risk for SCI mortality in the elderly males, as reported in other regions of China [12, 35]. The higher TBI and SCI mortality in males might have primarily resulted from the high mortality of motor vehicle crashes, falls and struck by or against which occurred more frequently in men than in women. Men might have more risk behaviors than women, including distracted driving because of cell phone uses [36], driving without helmets and seat belts, and alcohol consumption [37]. The high-risk behaviors of rural residents, insufficient injury prevention and medical care in rural areas could have contributed to the higher mortality in rural areas compared with urban areas [38, 39]. The elderly population were at an increased risk of having multiple comorbidities, which might lead to vision disorders, balance impairments, or cognitive impairment [22, 27].

In agreement with previous findings, motor vehicle crashes were the most common cause of TBI mortality in residents aged under 75 years [6] in Guangdong. However, in the US, firearm-related wounds dominated the cause of TBI mortality in young adults and the elderly [26]. In European countries, TBI mortality was mainly caused by falls [22]. Moreover, pedestrians aged 0–14 years and 65 years or greater were most vulnerable to TBI death, while motorcyclists yielded the highest rate of TBI death in the middle-aged population.

Similar to the findings in the US [40] and Australia [2], falls were the most important external cause for SCI mortality in all age groups, especially in the elderly. Motor vehicle crashes were an important cause of SCI death in the 24-65-year age group, which might be explained by the 8.5­year increase in life expectancy during the past two decades and the greater proportion of elderly people living with chronic diseases at risk of fall related injuries and death [41]. The elderly living without familial or social support might be at higher risk of disability or death due to delayed care for fall related injuries [42, 43].

### Implications

Our findings revealed a considerable burden of TBI caused by road traffic crashes in Guangdong province, highlighting the need for enhancing motor vehicle safety. First, prevention measures reported elsewhere should be adapted to the local region to ensure their acceptability, feasibility, and efficacy. Helmet use for bicyclists and motorcyclists, child restraint (car seat and booster seat use for child passengers), seat belt use for all vehicle passengers, and stringent traffic regulations should be enforced [6, 43]. Given the relatively high burden of falls leading to SCI, an integrated intervention should consist of medical and occupational therapy [44], professional environment hazards assessment and modification [45], vitamin D and calcium supplementation, hip protectors, reduction of the predisposing risk factors [46], shock-absorbing materials in children’s playgrounds, and periodic review of vision. Unfortunately, a number of interventions with proven efficacy to prevent major causes of injuries from motor vehicle crashes and falls have been underrepresented in Chinese laws and regulations [47]. Furthermore, effective interventions to improve the pre-hospital aid, hospital treatment, and rehabilitation services for TBI and SCI patients are needed [48, 49]. These may include developing a pre-hospital trauma care system by assimilating the experience from developed countries, speeding up efforts to ensure the individuals receive life-sustaining care immediately after injury, and instructing individuals in how to react in case of an injury.

### Study limitations and strengths

Our study was limited by a lack of robust injury incidence data. The data set of the DSP did not cover confounding factors including behavioral, rurality, environmental risk factors, TBI/SCI severity, pre-hospital and hospital care, which might have greatly influenced on the estimates of TBI and SCI mortality. Furthermore, TBI mortality might have been underestimated because of the missing S- or T-codes in injury-related deaths.

Despite these limitations, our study findings remain valid because the death registration in Guangdong province was based on the all-cause of death surveillance which covered all the residents residing in Guangdong. Data from the registration system aligned well with the vital registration data that achieved large increases in coverage over the past decade [50]. Moreover, we have used the empirical algorithm to minimize the risk of underestimation of the death surveillance. Finally, we tried to avoid misclassification bias by: 1) choosing the fatal and initial cause of death based on the death certificate and death survey when TBI and SCI occurred simultaneously; and 2) using subgroup analysis and multivariate negative binomial models to adjust for socio-demographic factors including location, sex, age and year.

## Conclusion

TBI and SCI are significant public health issues in Guangdong province. The TBI mortality remains stable, while SCI increases considerably since 2014. Males, rural residents, and the elderly are at increased risk of dying from TBI and SCI. The notable burden of TBI caused by road traffic crashes and the SCI caused by falls call for an urgent need to improve injury prevention, pre-hospital aid, hospital treatment and recovery.

## Supplementary Information

**Additional file 1: E-Table 1.** The internal data verification and the completeness of death surveillance. **E-Table 2.** Sample characteristics of the included Disease Surveillance Points of Guangdong province between 2014 and 2018. **E-Table 3.** Associations between the traumatic brain injury mortality from motor vehicle crashes and the socio-demographic variables, estimated with multivariate negative binomial regression model (Guangdong, China, 2014–2018). **E-Table 4.** Associations between spinal cord injury mortality from motor vehicle crashes and the socio-demographic variables, estimated with the multivariate negative binomial regression model (Guangdong, China, 2014–2018). **E-Figure 1.** Mortality rates from traumatic brain injury when stratified by location (urban/rural), sex, and age group in Guangdong, China, 2014–2018. (A) Stratification by sex; (B) stratification by age; (C) stratification by location group. Mortality rates in (A) and (C) were age-standardized based on the population of China in 2010. **E-Figure 2.** Mortality rates from spinal cord injury when stratified by sex, age and location (urban/rural) group in Guangdong China, 2014–2018. (A) Stratification by sex; (B) stratification by age; (C) stratification by location group. Mortality rates in (A) and (C) were age-standardized based on the population of China in 2010. **E-Figure 3.** Mortality rates from spinal cord injury due to motor vehicle crashes when stratified by urban/rural location, sex, and road user category (Guangdong, China, 2014–2018). (A) Male; (B) Female; (C) Urban areas; (D) Rural areas. Mortality rates were age-standardized based on the population of China in 2010.

## Data Availability

All data relevant to the study are included in the article or uploaded as supplementary information.

## Abbreviations

MRR: Mortality rate ratios
MVC: Motor vehicle crash
TBI: Traumatic brain injury
SCI: Spinal cord injury
