# Supplementary material for: Trends and external causes of traumatic brain injury and spinal cord injury mortality in south China, 2014–2018: an ecological study
Source: BMC Public Health. 2021 Dec 7;21:2225. doi: 10.1186/s12889-021-12225-2 (PMC8653562; doi:10.1186/s12889-021-12225-2)
Supplement: Supplementary file 1 — Additional file 1: E-Table 1. The internal data verification and the completeness of death surveillance. E-Table 2. Sample characteristics of the included Disease Surveillance Points of Guangdong province between 2014 and 2018. E-Table 3. Associations between the traumatic brain injury mortality from motor vehicle crashes and the socio-demographic variables, estimated with multivariate negative binomial regression model (Guangdong, China, 2014–2018). E-Table 4. Associations between spinal cord injury mortality from motor vehicle crashes and the socio-demographic variables, estimated with the multivariate negative binomial regression model (Guangdong, China, 2014–2018). E-Figure 1. Mortality rates from traumatic brain injury when stratified by location (urban/rural), sex, and age group in Guangdong, China, 2014–2018. (A) Stratification by sex; (B) stratification by age; (C) stratification by location group. Mortality rates in (A) and (C) were age-standardized based on the population of China in 2010. E-Figure 2. Mortality rates from spinal cord injury when stratified by sex, age and location (urban/rural) group in Guangdong China, 2014–2018. (A) Stratification by sex; (B) stratification by age; (C) stratification by location group. Mortality rates in (A) and (C) were age-standardized based on the population of China in 2010. E-Figure 3. Mortality rates from spinal cord injury due to motor vehicle crashes when stratified by urban/rural location, sex, and road user category (Guangdong, China, 2014–2018). (A) Male; (B) Female; (C) Urban areas; (D) Rural areas. Mortality rates were age-standardized based on the population of China in 2010. [file 12889_2021_12225_MOESM1_ESM.docx]

**Online Supplement**

**Trends and external causes of traumatic brain injury and spinal cord injury mortality in southern China, 2014–2018: An ecological study**

Xue-yan Zheng^1*^, Qian Yi^1,2*^, Xiao-jun Xu^1*^, Rui-lin Meng, Shu-li Ma^2^, Si-li Tang^3^, Hao-feng Xu^1^, Ying-shan Xu^1^，Yan-jun Xu^1#^, Yi Yang^2#^

1 Guangdong provincial center for disease control and prevention, Guangdong, China.

2 Department of Epidemiology and Biostatistics, School of Public Health, Guangdong Pharmaceutical University, Guangzhou, China.

3 School of Public Health, Southern Medical University, Guangdong, China

**^*^** These authors contributed equally to this work

^#^ These authors are joint senior authors on this work.

^#^ **Correspondence author 1:** **Yan-jun Xu.** Guangdong provincial center for disease control and prevention, Guangdong, China. Address: 160 Qunxian Road, Panyu district, Guangzhou, Guangdong, China. Fax: 020-31051492, Phone: 020-31051353, E-mail:[759888406@qq.com](mailto:759888406@qq.com).

**Correspondence author 2:** **Yi Yang.** Department of Epidemiology and Biostatistics, School of Public Health, Guangdong Pharmaceutical University, Guangzhou, China. E-mail: yangyigz@163.com

**Methods**

***Completeness of death surveillance***

The completeness of death surveillance data was estimated empirically according to the methods proposed by Adair and Lopez [1]. Briefly, the following equation was calculated to predict the completeness:

logit$($𝐶^All^$)$=𝑅eg𝐶𝐷𝑅𝑠𝑞×(-0.0238$)$+𝑅eg𝐶𝐷𝑅×0.8419+%65×(-19.6118)+ln(5q0$)$×(-1.5135$)$+𝑌𝑒𝑎𝑟×(-0.0251$)$+44.3755+*γ*

where $C^{A\text{ll}}$ was the completeness of registration of all ages, $\log it\left( C^{A\text{ll}} \right)$ denoted$\text{ ln}\left( \frac{C^{\text{All}}}{1\text{-}\text{C}^{\text{All}}} \right)$, $R\text{eg}CDR$ was the registered crude death rate (CDR), $R\text{eg}CDR\text{sq}$ was the square of *RegCDR*, %65 was the fraction of the population aged 65 years or greater (%), $\text{ln}\left( 5q0 \right)$ was the natural logarithm of the under-5 mortality rate, *Year* was the calendar year, *γ* was the random effect, and the predicted completeness was converted by using the inverse logit: $\frac{e^{\text{logit}\left( C^{A\text{ll}} \right)}}{e^{\text{logit}\left( C^{A\text{ll}} \right)}+1}$. The results of the internal data verification and the completeness of death surveillance for each district in this study were listed in **E-Table 1.**


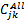

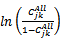

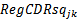

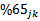

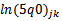


**E-Table 1. The internal data verification and the completeness of death surveillance**

| **District** | **2014** | | | |  | **2015** | | | |  | **2016** | | | |  | **2017** | | | |  | **2018** | | | |
| --- | --- | --- | --- | --- | --- | --- | --- | --- | --- | --- | --- | --- | --- | --- | --- | --- | --- | --- | --- | --- | --- | --- | --- | --- |
|  | **CDR** | **%65** | **5*q*0** | ***C^All^*** |  | **CDR** | **%65** | **5*q*0** | ***C^All^*** |  | **CDR** | **%65** | **5*q*0** | ***C^All^*** |  | **CDR** | **%65** | **5*q*0** | ***C^All^*** |  | **CDR** | **%65** | **5*q*0** | ***C^All^*** |
| Liwan | 8.7 | 15.7 | 6.2 | 98.1 |  | 8.1 | 19.1 | 3.4 | 98.0 |  | 8.4 | 17.1 | 4.0 | 98.5 |  | 8.4 | 17.4 | 3.8 | 98.5 |  | 8.5 | 17.8 | 2.2 | 99.3 |
| Yuexiu | 6.8 | 14.8 | 3.9 | 98.1 |  | 6.4 | 17.7 | 4.2 | 95.4 |  | 7.0 | 15.8 | 4.5 | 97.3 |  | 6.8 | 16.3 | 4.0 | 97.1 |  | 7.2 | 16.7 | 3.3 | 98.1 |
| Panyu | 4.8 | 8.7 | 5.4 | 97.1 |  | 4.2 | 10.4 | 5.4 | 94.3 |  | 4.4 | 9.2 | 6.0 | 95.2 |  | 4.3 | 9.3 | 2.1 | 98.8 |  | 4.3 | 9.3 | 4.0 | 96.8 |
| Wujiang | 5.9 | 13.1 | 13.3 | 86.9 |  | 5.6 | 13.6 | 15.6 | 80.5 |  | 5.3 | 10.9 | 9.2 | 92.4 |  | 5.5 | 11.0 | 4.9 | 97.2 |  | 5.6 | 11.2 | 6.1 | 96.2 |
| Wengyuan | 6.0 | 9.3 | 10.2 | 95.8 |  | 5.7 | 12.2 | 17.1 | 83.0 |  | 5.9 | 9.6 | 7.3 | 97.1 |  | 5.9 | 9.4 | 7.0 | 97.2 |  | 5.9 | 9.6 | 5.7 | 97.9 |
| Nanxiong | 6.4 | 9.4 | 8.1 | 97.6 |  | 5.8 | 12.8 | 17.2 | 81.8 |  | 5.7 | 9.4 | 8.1 | 96.4 |  | 6.2 | 9.7 | 7.1 | 97.4 |  | 6.5 | 10.0 | 7.0 | 97.7 |
| Nanshan | 1.1 | 2.9 | 3.3 | 94.1 |  | 0.9 | 3.4 | 5.1 | 86.5 |  | 0.8 | 3.1 | 2.3 | 95.4 |  | 0.9 | 3.5 | 2.0 | 96.0 |  | 1.0 | 3.5 | 1.7 | 97.0 |
| Doumen | 5.7 | 9.7 | 10.9 | 94.3 |  | 4.9 | 11.1 | 7.7 | 92.8 |  | 5.1 | 9.4 | 6.5 | 96.1 |  | 5.0 | 9.6 | 4.7 | 97.3 |  | 4.9 | 9.8 | 3.9 | 97.7 |
| Chancheng | 5.9 | 13.1 | 9.8 | 91.6 |  | 5.9 | 15.5 | 12.0 | 82.7 |  | 6.3 | 13.9 | 7.1 | 94.7 |  | 5.8 | 13.9 | 5.1 | 95.7 |  | 5.7 | 13.9 | 4.7 | 95.8 |
| Shunde | 6.1 | 10.8 | 6.9 | 97.1 |  | 5.6 | 12.6 | 6.7 | 94.7 |  | 5.6 | 11.0 | 5.1 | 97.3 |  | 5.6 | 11.2 | 3.9 | 98.0 |  | 5.2 | 11.3 | 3.9 | 97.5 |
| Taishan | 8.0 | 11.6 | 6.0 | 98.9 |  | 7.3 | 16.0 | 11.7 | 90.8 |  | 7.9 | 13.6 | 7.3 | 97.7 |  | 7.8 | 14.1 | 6.9 | 97.5 |  | 7.6 | 14.5 | 4.0 | 98.6 |
| Heshan | 7.5 | 10.8 | 5.3 | 99.0 |  | 6.5 | 16.2 | 5.8 | 94.8 |  | 7.1 | 13.6 | 7.0 | 96.7 |  | 7.3 | 13.3 | 5.6 | 97.9 |  | 7.1 | 13.6 | 5.3 | 97.7 |
| Wuchuan | 4.0 | 9.5 | 6.6 | 92.6 |  | 3.1 | 10.4 | 20.8 | 49.2 |  | 3.4 | 8.4 | 12.2 | 80.1 |  | 3.9 | 8.5 | 10.5 | 86.8 |  | 4.1 | 8.7 | 12.4 | 84.6 |
| Gaozhou | 3.4 | 10.1 | 18.5 | 61.8 |  | 3.2 | 11.5 | 31.8 | 31.1 |  | 3.8 | 9.8 | 17.0 | 70.8 |  | 4.3 | 9.6 | 14.2 | 81.4 |  | 4.4 | 9.8 | 14.3 | 81.2 |
| Duanzhou | 5.3 | 12.0 | 14.3 | 84.5 |  | 5.2 | 13.6 | 9.2 | 87.5 |  | 5.2 | 11.9 | 6.6 | 94.2 |  | 5.2 | 11.6 | 6.6 | 94.2 |  | 5.2 | 11.7 | 3.5 | 97.7 |
| Sihui | 7.0 | 8.7 | 7.6 | 98.6 |  | 6.0 | 14.2 | 9.0 | 91.3 |  | 6.6 | 11.8 | 5.0 | 98.2 |  | 6.5 | 11.7 | 4.8 | 98.2 |  | 6.3 | 11.9 | 4.6 | 98.0 |
| Huiyang | 5.4 | 8.1 | 7.9 | 96.9 |  | 5.3 | 8.0 | 10.4 | 94.9 |  | 5.7 | 6.1 | 6.3 | 98.6 |  | 6.6 | 6.4 | 7.1 | 98.9 |  | 5.5 | 6.5 | 6.5 | 98.2 |
| Boluo | 5.5 | 9.1 | 14.2 | 91.5 |  | 4.7 | 11.5 | 11.6 | 84.7 |  | 5.0 | 9.5 | 8.8 | 93.5 |  | 5.1 | 9.3 | 5.9 | 96.7 |  | 5.3 | 9.4 | 7.2 | 95.8 |
| Wuhua | 5.5 | 7.8 | 9.1 | 96.6 |  | 3.9 | 8.4 | 18.7 | 75.2 |  | 3.8 | 7.2 | 10.0 | 89.9 |  | 4.2 | 7.1 | 7.5 | 94.6 |  | 4.3 | 7.3 | 8.3 | 93.8 |
| Shanwei | 5.7 | 6.2 | 5.7 | 98.8 |  | 6.5 | 5.9 | 27.9 | 92.4 |  | 6.5 | 4.7 | 15.6 | 97.4 |  | 4.9 | 5.1 | 5.6 | 98.4 |  | 3.9 | 5.3 | 5.6 | 96.9 |
| Heping | 5.4 | 26.0 | 3.2 | 78.5 |  | 3.9 | 11.7 | 20.7 | 57.8 |  | 5.1 | 9.5 | 13.5 | 89.4 |  | 5.1 | 9.4 | 9.6 | 93.3 |  | 5.4 | 9.5 | 5.9 | 96.9 |
| Yangdong | 5.0 | 10.7 | 4.0 | 97.6 |  | 4.6 | 12.6 | 13.0 | 77.6 |  | 5.3 | 10.7 | 11.1 | 90.5 |  | 5.1 | 11.0 | 6.2 | 95.2 |  | 5.1 | 11.3 | 6.9 | 93.8 |
| Qingcheng | 5.8 | 9.7 | 10.2 | 95.0 |  | 4.8 | 12.3 | 9.9 | 86.5 |  | 5.3 | 9.9 | 7.2 | 95.6 |  | 5.2 | 10.0 | 3.3 | 98.5 |  | 5.6 | 10.2 | 5.6 | 97.1 |
| Yingde | 5.6 | 11.0 | 12.0 | 91.0 |  | 4.8 | 12.4 | 25.3 | 60.7 |  | 5.2 | 9.3 | 12.1 | 91.5 |  | 5.3 | 9.3 | 5.4 | 97.5 |  | 5.4 | 9.4 | 5.6 | 97.4 |
| Zhongshan | 5.9 | 9.8 | 7.0 | 97.3 |  | 5.6 | 12.4 | 9.3 | 91.9 |  | 5.8 | 10.4 | 6.0 | 97.2 |  | 5.6 | 10.5 | 5.0 | 97.5 |  | 5.4 | 10.6 | 3.8 | 98.0 |
| Huilai | 2.7 | 8.9 | 2.9 | 95.2 |  | 2.6 | 6.0 | 12.7 | 78.3 |  | 2.9 | 5.3 | 14.9 | 79.5 |  | 2.7 | 5.5 | 7.5 | 89.8 |  | 3.1 | 5.7 | 8.3 | 90.3 |
| Yuncheng | 4.7 | 10.9 | 7.2 | 93.0 |  | 5.2 | 12.7 | 14.0 | 81.9 |  | 5.5 | 10.2 | 6.9 | 96.1 |  | 5.5 | 10.1 | 6.4 | 96.6 |  | 5.7 | 10.3 | 8.0 | 95.4 |
| Luoding | 4.6 | 8.4 | 7.7 | 94.9 |  | 4.8 | 8.6 | 13.2 | 89.5 |  | 5.3 | 6.2 | 12.2 | 95.3 |  | 5.1 | 6.4 | 10.6 | 95.4 |  | 4.6 | 6.6 | 7.1 | 96.3 |

Note: CDR, the registered crude death rate (%); %65, the fraction of the population aged 65 years or greater (%); 5*q*0, the under-5 mortality rate (per 10^5^ population); *C^All^*, the completeness of registration of all ages (%).

**Reference:**

1. Adair T, Lopez AD. Estimating the completeness of death registration: An empirical method. PLoS One. 2018; 13(5):e0197047.

**Results**

**E-Table 2. Sample characteristics of the included Disease Surveillance Points of Guangdong province between 2014 and 2018**

| **Demographic characteristic** | **2014** | |  | **2015** | |  | **2016** | |  | **2017** | |  | **2018** | |  | **China census population in 2010** | |
| --- | --- | --- | --- | --- | --- | --- | --- | --- | --- | --- | --- | --- | --- | --- | --- | --- | --- |
|  | **No.** | **%** |  | **No.** | **%** |  | **No.** | **%** |  | **No.** | **%** |  | **No.** | **%** |  | **No.** | **%** |
| Sex |  |  |  |  |  |  |  |  |  |  |  |  |  |  |  |  |  |
| Male | 11,515,312 | 51.5 |  | 12,366,035 | 51.4 |  | 12,383,069 | 51.2 |  | 12,443,215 | 51.1 |  | 12,563,746 | 51.0 |  | 682,329,104 | 51.2 |
| Female | 10,861,589 | 48.5 |  | 11,706,783 | 48.6 |  | 11,807,802 | 48.8 |  | 11,918,384 | 48.9 |  | 12,058,675 | 49.0 |  | 650,481,765 | 48.8 |
| Age group |  |  |  |  |  |  |  |  |  |  |  |  |  |  |  |  |  |
| 0~4years | 1,273,339 | 5.7 |  | 1,147,357 | 4.8 |  | 1,304,650 | 5.4 |  | 1,443,642 | 5.9 |  | 1,476,637 | 6.0 |  | 75,532,610 | 5.7 |
| 5~14years | 2,674,862 | 12.0 |  | 2,849,647 | 11.8 |  | 2,719,130 | 11.2 |  | 2,898,834 | 11.9 |  | 2,981,411 | 12.1 |  | 145,790,011 | 10.9 |
| 15~24years | 3,595,491 | 16.1 |  | 4,270,641 | 17.7 |  | 3,554,008 | 14.7 |  | 3,424,105 | 14.1 |  | 3,344,139 | 13.6 |  | 227,301,632 | 17.1 |
| 25~44years | 7,099,156 | 31.7 |  | 7,530,847 | 31.3 |  | 8,173,184 | 33.8 |  | 8,004,756 | 32.9 |  | 8,074,879 | 32.8 |  | 440,931,978 | 33.1 |
| 45~64years | 5,417,600 | 24.2 |  | 5,486,329 | 22.8 |  | 6,105,300 | 25.2 |  | 6,221,987 | 25.5 |  | 6,305,534 | 25.6 |  | 324,327,480 | 24.3 |
| 65~74years | 1,312,118 | 5.9 |  | 1,348,366 | 5.6 |  | 1,315,452 | 5.4 |  | 1,396,119 | 5.7 |  | 1,457,186 | 5.9 |  | 74,085,679 | 5.6 |
| ≥75years | 1,004,335 | 4.5 |  | 1,439,631 | 6.0 |  | 1,019,147 | 4.2 |  | 972,156 | 4.0 |  | 982,635 | 4.0 |  | 44,841,479 | 3.4 |
| Location |  |  |  |  |  |  |  |  |  |  |  |  |  |  |  |  |  |
| Urban | 9,772,211 | 43.7 |  | 10,342,139 | 43.0 |  | 10,528,416 | 43.5 |  | 10,775,153 | 44.2 |  | 10,990,591 | 44.6 |  | 670,005,546 | 50.3 |
| Rural | 12,604,690 | 56.3 |  | 13,730,679 | 57.0 |  | 13,662,455 | 56.5 |  | 13,586,446 | 55.8 |  | 13,631,830 | 55.4 |  | 662,805,323 | 49.7 |
| Total | 22,376,901 | 100.0 |  | 24,072,818 | 100.0 |  | 24,190,871 | 100.0 |  | 24,361,599 | 100.0 |  | 24,622,421 | 100.0 |  | 1,242,612,226 | 100.0 |

**E-Table 3. Associations between the traumatic brain injury mortality from motor vehicle crashes and the socio-demographic variables, estimated with multivariate negative binomial regression model (Guangdong, China, 2014-2018).**

| **Socio-demographic variable** | **TBI mortality from motor vehicle crashes by road user category** | | | | | | | | | | | | | | | | | | |
| --- | --- | --- | --- | --- | --- | --- | --- | --- | --- | --- | --- | --- | --- | --- | --- | --- | --- | --- | --- |
|  | **Vehicle occupant** | | |  | **Motorcyclist** | | |  | **Pedal cyclist** | | |  | **Pedestrian** | | |  | **All others** | | |
|  | ***MRR*** | **95%*CI*** | |  | ***MRR*** | **95%*CI*** | |  | ***MRR*** | **95%*CI*** | |  | ***MRR*** | **95%*CI*** | |  | ***MRR*** | **95%*CI*** | |
|  |  | **Lower** | **Upper** |  |  | **Lower** | **Upper** |  |  | **Lower** | **Upper** |  |  | **Lower** | **Upper** |  |  | **Lower** | **Upper** |
| **Sex (reference= female)** | | |  |  |  |  |  |  |  |  |  |  |  |  |  |  |  |  |  |
| Male | 2.3* | 1.3 | 3.8 |  | 4.6* | 3.2 | 6.7 |  | 2.7* | 1.3 | 5.4 |  | 2.0* | 1.6 | 2.5 |  | 2.6* | 1.7 | 3.9 |
| **Age (reference= 25~44 years)** | | |  |  |  |  |  |  |  |  |  |  |  |  |  |  |  |  |  |
| 0~4 | 0.4* | 0.2 | 1.1 |  | 0.2* | 0.1 | 0.4 |  | 0.1* | 0.0 | 0.5 |  | 0.9* | 0.6 | 1.4 |  | 0.5* | 0.2 | 1.0 |
| 5~14 | 0.1* | 0.0 | 0.2 |  | 0.2* | 0.1 | 0.3 |  | 0.3* | 0.1 | 1.0 |  | 0.4* | 0.3 | 0.6 |  | 0.3* | 0.1 | 0.6 |
| 15~24 | 0.6* | 0.3 | 1.6 |  | 1.0* | 0.5 | 2.0 |  | 0.6* | 0.2 | 1.9 |  | 0.9* | 0.6 | 1.4 |  | 1.0* | 0.5 | 2.0 |
| 45~64 | 1.1* | 0.5 | 2.9 |  | 1.6* | 0.8 | 3.2 |  | 3.7* | 1.2 | 11.8 |  | 1.9* | 1.3 | 2.9 |  | 1.7* | 0.8 | 3.6 |
| 65~74 | 0.9* | 0.4 | 2.3 |  | 1.5* | 0.8 | 3.1 |  | 7.6* | 2.3 | 25.1 |  | 4.5* | 2.9 | 6.8 |  | 2.9* | 1.4 | 6.2 |
| ≥75 | 0.8* | 0.3 | 2.1 |  | 0.7* | 0.4 | 1.4 |  | 6.6* | 1.9 | 22.6 |  | 7.2* | 4.7 | 11.0 |  | 3.1* | 1.5 | 6.7 |
| **Location (reference= urban)** | | |  |  |  |  |  |  |  |  |  |  |  |  |  |  |  |  |  |
| Rural | 2.1* | 1.2 | 3.4 |  | 3.3* | 2.3 | 4.7 |  | 4.1* | 2.1 | 8.3 |  | 2.9* | 2.3 | 3.6 |  | 1.3* | 0.9 | 2.0 |
| **Year (reference= 2014)** | | |  |  |  |  |  |  |  |  |  |  |  |  |  |  |  |  |  |
| 2015 | 2.4* | 1.1 | 5.2 |  | 1.3* | 0.7 | 2.3 |  | 1.0* | 0.4 | 2.8 |  | 1.0* | 0.7 | 1.5 |  | 1.7* | 0.9 | 3.1 |
| 2016 | 1.7* | 0.8 | 3.8 |  | 1.3* | 0.7 | 2.3 |  | 1.1* | 0.4 | 3.0 |  | 0.8* | 0.6 | 1.1 |  | 1.6* | 0.8 | 3.0 |
| 2017 | 1.8* | 0.8 | 4.1 |  | 1.1* | 0.6 | 2.0 |  | 1.5* | 0.6 | 4.3 |  | 1.0* | 0.7 | 1.4 |  | 1.3* | 0.7 | 2.4 |
| 2018 | 1.1* | 0.5 | 2.5 |  | 0.9* | 0.5 | 1.7 |  | 1.3* | 0.5 | 3.8 |  | 0.7* | 0.5 | 1.0 |  | 1.5* | 0.8 | 2.8 |

**P* < 0.05; *MRR*: mortality rate ratio, adjusted for location, sex, age group and year; *CI*: Confidence interval; MRR, mortality rate ratio; TBI: traumatic brain injury.

**E-Table 4. Associations between spinal cord injury mortality from motor vehicle crashes and the socio-demographic variables, estimated with the multivariate negative binomial regression model (Guangdong, China, 2014-2018).**

| **Socio-demographic variable** | **SCI mortality from motor vehicle crashes by road user category** | | | | | | | | | | |
| --- | --- | --- | --- | --- | --- | --- | --- | --- | --- | --- | --- |
|  | **Vehicle occupant** | | |  | **Motorcyclist** | | |  | **Others** | | |
|  | ***MRR*** | **95%*CI*** | |  | ***MRR*** | **95%*CI*** | |  | ***MRR*** | **95%*CI*** | |
|  |  | **Lower** | **Upper** |  |  | **Lower** | **Upper** |  |  | **Lower** | **Upper** |
| **Sex (reference= female)** |  | | | | | | | | | | |
| Male | 6.7* | 0.8 | 99.4 |  | 16.0* | 1.9 | 133.7 |  | 73.2 | 1.2 | 73.3 |
| **Age (reference= 25~44 years)** |  | | | | | | | | | | |
| 0~4 | 0.0* | 0.0 | 0.0 |  | 0.1* | 0.0 | 2.7 |  | 0.0 | 0.0 | 0.0 |
| 5~14 | 0.0* | 0.0 | 0.0 |  | 0.1* | 0.0 | 1.8 |  | 0.0 | 0.0 | 0.0 |
| 15~24 | 0.0* | 0.0 | 0.1 |  | 0.0* | 0.0 | 0.0 |  | 0.0 | 0.0 | 0.0 |
| 45~64 | 0.9* | 0.1 | 18.3 |  | 50.4* | 1.5 | 2306.2 |  | 3.1 | 2.9 | 3.5 |
| 65~74 | 1.0* | 0.0 | 27.7 |  | 0.4* | 0.0 | 9.1 |  | 3.6 | 3.5 | 3.9 |
| ≥75 | 0.0* | 0.0 | 1.0 |  | 8.6* | 0.4 | 204.0 |  | 169.6 | 1.0 | 49486.6 |
| **Location (reference= urban)** |  |  |  |  |  |  |  |  |  |  |  |
| Rural | 2.1* | 0.2 | 20.0 |  | 30.7* | 3.9 | 252.2 |  | 0.0 | 0.0 | 2.4 |
| **Year (reference= 2014)** |  | | | | | | | | | | |
| 2015 | 1.5* | 0.1 | 51.8 |  | 17.2* | 1.0 | 315.6 |  | 0.0 | 0.0 | 5.1 |
| 2016 | 0.5* | 0.0 | 14.3 |  | 0.1* | 0.0 | 1.6 |  | 291.9 | 1.4 | 292.1 |
| 2017 | 1.8* | 0.1 | 34.2 |  | 0.8* | 0.1 | 21.1 |  | 3.3 | 0.0 | 3.8 |
| 2018 | 215.7* | 4.3 | 9872.8 |  | 0.9* | 0.1 | 14.4 |  | 232.7 | 0.6 | 97097.1 |

**P* < 0.05; *MRR*: mortality rate ratio, adjusted for location, sex, age group and year; *CI*: Confidence interval; MRR, mortality rate ratio; SCI: spinal cord injury.

**
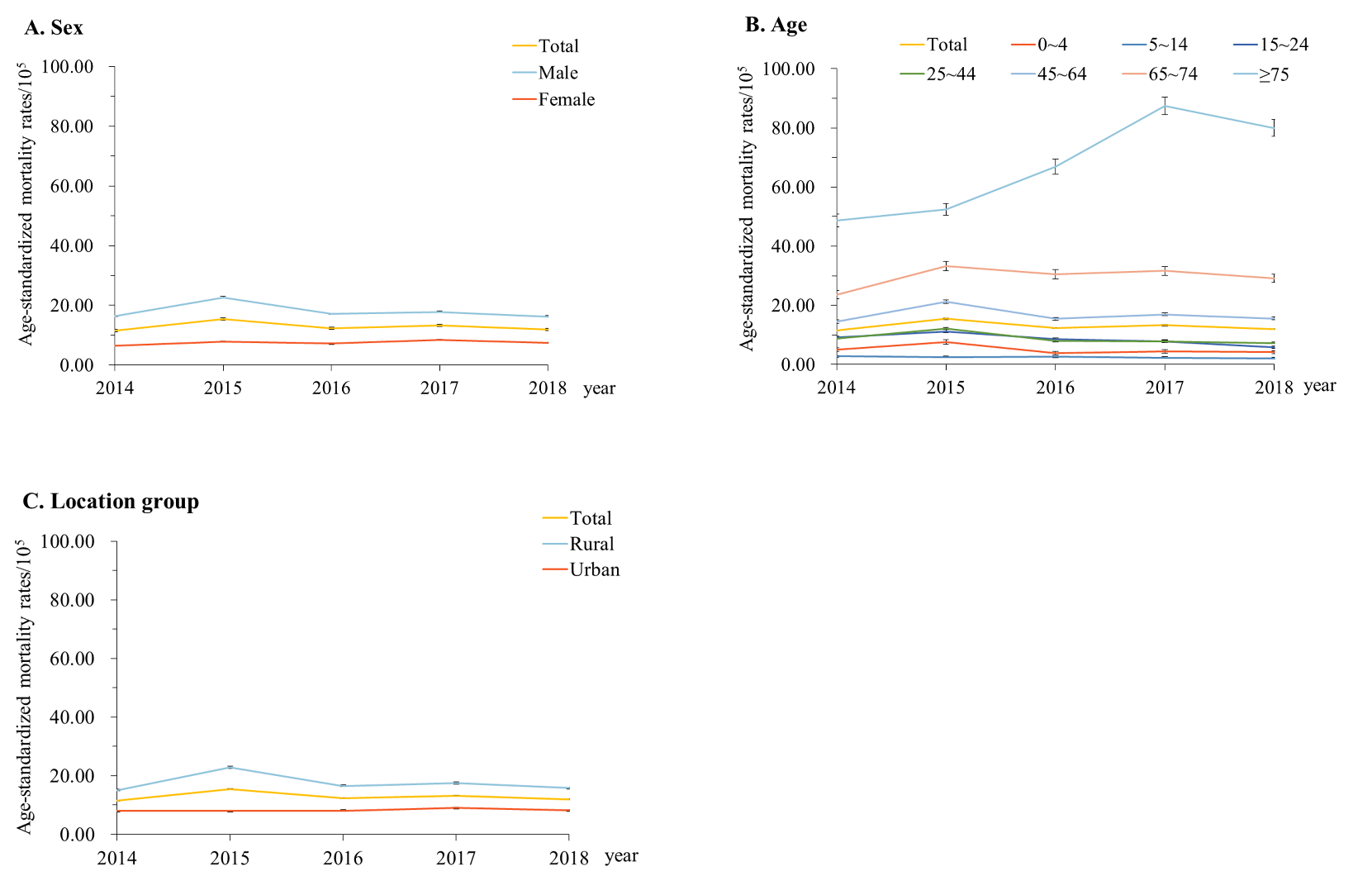
**

**E-Figure 1. Mortality rates from traumatic brain injury when stratified by location (urban/rural), sex, and age group in Guangdong, China, 2014-2018. (A) Stratification by sex; (B) stratification by age; (C) stratification by location group. Mortality rates in (A) and (C) were age-standardized based on the population of China in 2010.**

**
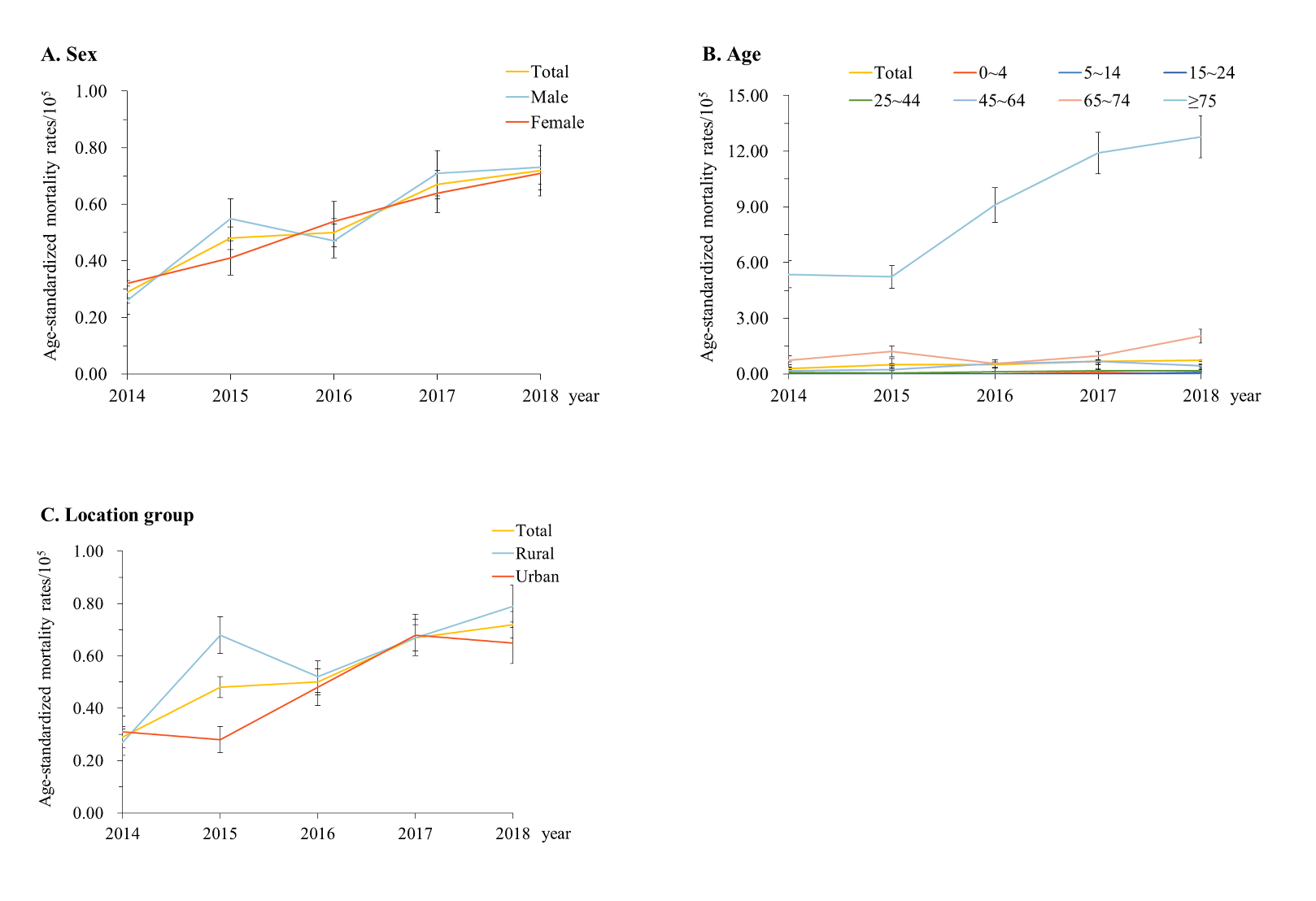
**

**E-Figure 2. Mortality rates from spinal cord injury when stratified by sex, age and location (urban/rural) group in Guangdong China, 2014-2018. (A) Stratification by sex; (B) stratification by age; (C) stratification by location group. Mortality rates in (A) and (C) were age-standardized based on the population of China in 2010.**

**
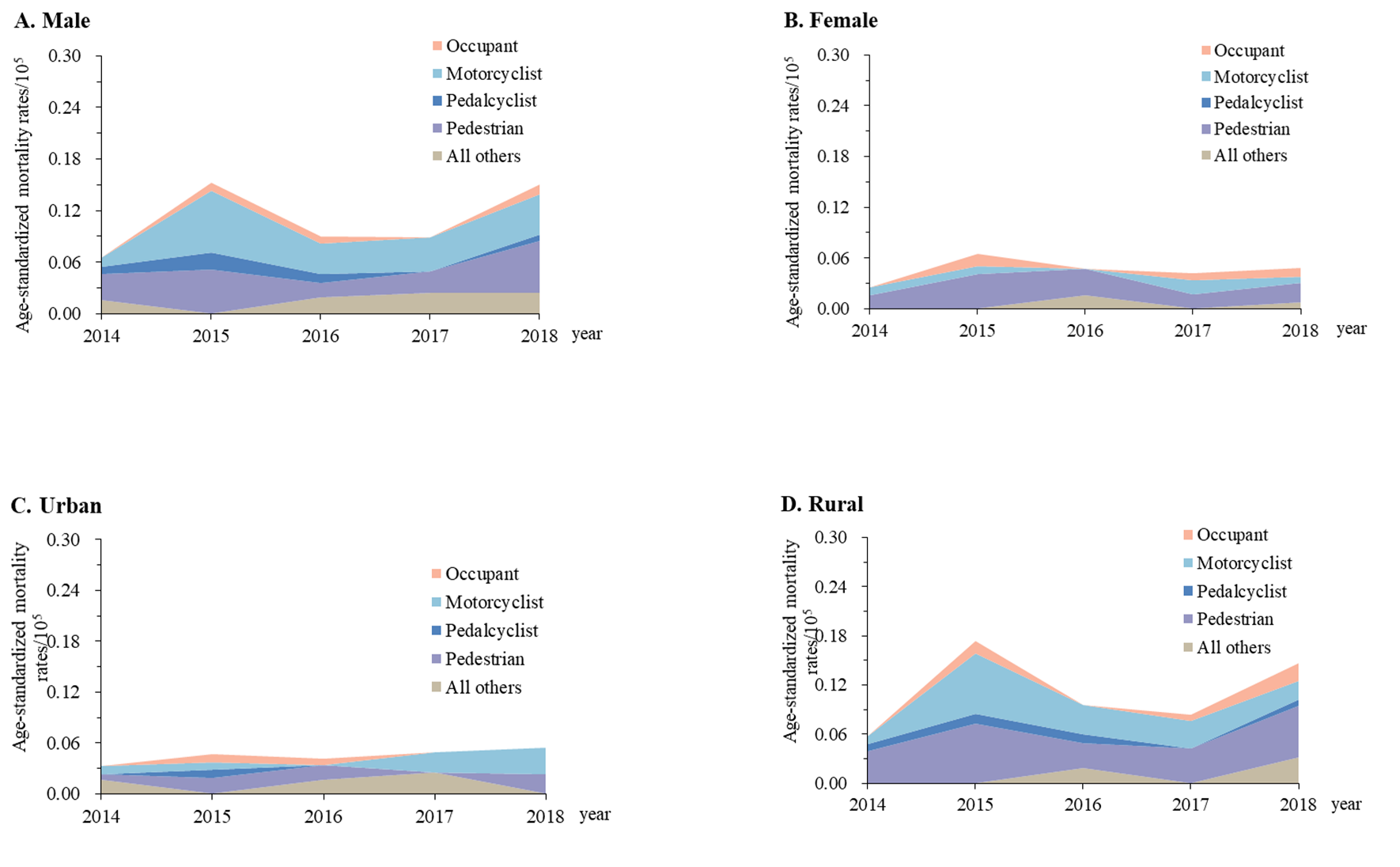
**

**E-Figure 3. Mortality rates from spinal cord injury due to motor vehicle crashes when stratified by urban/rural location, sex, and road user category (Guangdong, China, 2014–2018). (A) Male; (B) female; (C) Urban areas; (D) rural areas. Mortality rates were age-standardized based on the population of China in 2010**
